# Naphthalenes and Quinolines by Domino Reactions of Morita–Baylis–Hillman Acetates

**DOI:** 10.3390/molecules25215168

**Published:** 2020-11-06

**Authors:** Joel K. Annor-Gyamfi, Ebenezer Ametsetor, Kevin Meraz, Richard A. Bunce

**Affiliations:** Department of Chemistry, Oklahoma State University, Stillwater, OK 74078–3071, USA; jannorg@okstate.edu (J.K.A.-G.); eametse@okstate.edu (E.A.); meraz.kevin@outlook.com (K.M.)

**Keywords:** Morita–Baylis–Hillman acetates, active methylene compounds, domino reactions, naphthalenes, quinolines

## Abstract

An efficient synthetic route to highly functionalized naphthalenes and quinolines has been developed using domino reactions between Morita–Baylis–Hillman (MBH) acetates and active methylene compounds (AMCs) promoted by anhydrous K_2_CO_3_ in dry *N*,*N*-dimethylformamide (DMF) at 23 °C. The substrates incorporate allylic acetates positioned adjacent to a Michael acceptor as well as an aromatic ring activated toward a S_N_Ar ring closure. A control experiment indicated that the initial reaction was an S_N_2’-type displacement of a side chain acetoxy by the AMC anion to afford the alkene product bearing the added nucleophile *trans* to the S_N_Ar aromatic ring acceptor. Thus, equilibration of the alkene geometry of the initial product was required prior to cyclization. Products were isolated in good to excellent yields. Numerous cases (24) are reported, and several mechanistic possibilities are discussed.

## 1. Introduction

One facet of our research program has focused on domino reactions for the rapid assembly of molecules with potential use in drug synthesis. This requires highly functionalized compounds with reactive sites strategically positioned to capture intermediates from an initial reaction in one or more subsequent reactions to produce targets of high value in an efficient, eco-friendly manner. Among the compounds meeting these requirements are the products of the Morita–Baylis–Hillman (MBH) reaction. These highly functionalized adducts have proven quite useful in drug synthesis [1,2]. Additionally, the naphthalenes [3] and quinolines [4,5] targeted in this work could have immense value in medicinal chemistry. 

**Figure 1:**
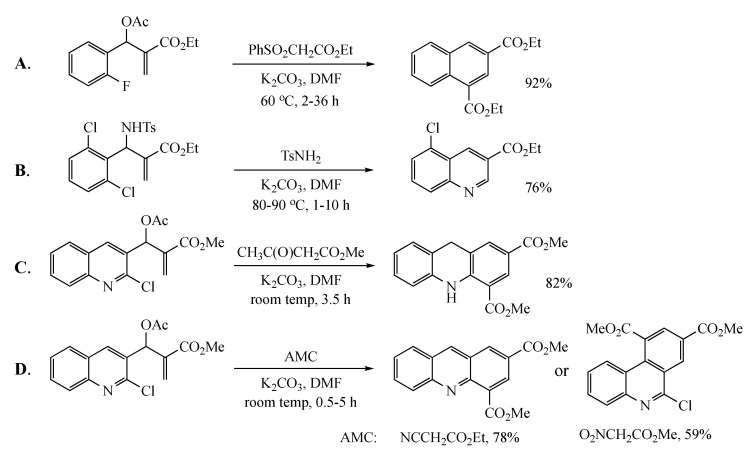
Previous cyclizations to generate aromatic systems using Morita–Baylis–Hillman (MBH) acetates. (**A**): Formation of naphthalenes; (**B**): Formation of quinolines; (**C**): Formation of dihydroacridines; (**D**): Formation of acridines and phenanthridines.

Previous work by others has appeared in this area, but details were lacking, and the diversity of examples reported was limited. One article outlined the formation of a number of naphthalene derivatives [6] from MBH acetates and active methylene compounds (AMCs) (Figure 1A), but the initial adduct was assumed to have the correct geometry for ring closure, and the only molecule eliminated during the final aromatization was benzenesulfinic acid (PhSO_2_H). A second report described a synthesis of 3-quinolinecarboxylic esters [7] involving the reaction of ethyl 2-((2-chlorophenyl)((tosylsulfonamido)methyl)acrylate acrylate with tosylsulfonamide (Figure 1B). A third study [8] advanced a synthesis of dihydroacridines from MBH acetates derived from 2-chloroquinoline-3-carboxaldehyde and AMCs to fuse a substituted benzene ring to the heterocycle (Figure 1C). The final account detailed the AMC-dependent annulation of substituted benzene rings by reaction of this same MBH precursor to give acridines and phenanthridines [9] (Figure 1D). The current work describes the synthesis of naphthalenes and quinolines with a broader range of functionality, evaluates a selection of different leaving groups, and discusses several mechanistic possibilities.

## 2. Results and Discussion 

**Scheme 1:**
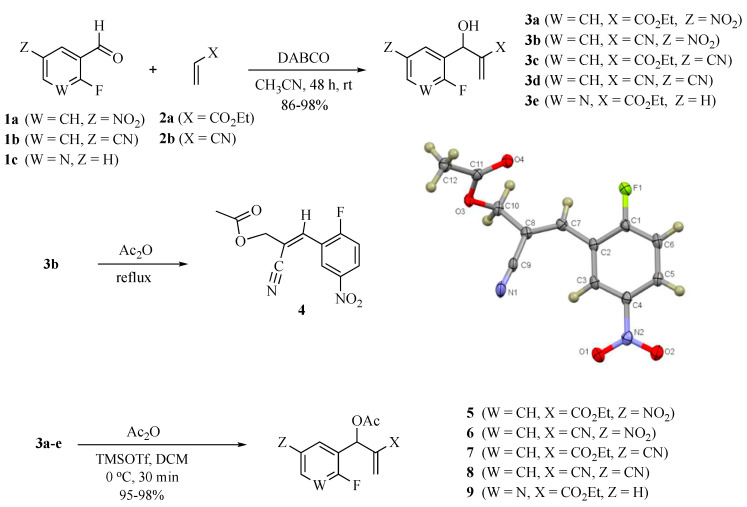
Synthesis of MBH acetates for cyclization and rearranged product **4** (CCDC 2035023).

The MBH reaction was first reported in 1968 [10] and has undergone many modifications [11,12,13] to improve its outcome. The generally accepted procedure calls for 1 equiv. of the aldehyde and 1.2 equiv. of the electron-poor alkene in the presence of 1.5 equiv. of 1,4-diazabicyclo [2.2.2]octane (DABCO) in acetonitrile (ACN) at room temperature (23 °C) [14]. For the current project, we have generated these adducts by the reaction of polarized alkenes with aromatic aldehydes incorporating functionality that further activates the aromatic ring toward a S_N_Ar reaction using a 2:1 stoichiometry of alkene:aldehyde with 1.2 equiv. of DABCO (Scheme 1). Thus, 2-fluoro-5-nitrobenzaldehyde (**1a**), 2-fluoro-5-cyanobenzaldehyde (**1b**), and 2-fluoronicotinaldehyde (**1c**) were each treated with ethyl acrylate (**2a**) and acrylonitrile (**2b**) in ACN at 23 °C for 2 days to give the MBH alcohols (**3a**–**e**) in 86–98% yields. Attempts to acylate the alcohol adduct **3b** with acetic anhydride at reflux led to acylation of the alcohol and allylic inversion of the acetate to afford (*E*)-2-cyano-3-(2-fluoro-5-nitrophenyl)allyl acetate (**4**). Interestingly, an X-ray structure of this product showed the acetoxymethyl group of the rearranged acetate to be *trans* to the substrate aromatic ring (see Scheme 1 and the Supplementary Materials). Acylation of the MBH adducts without rearrangement was achieved using trimethylsilyl trifluoromethanesulfonate (TMSOTf) as an acylation catalyst [15] in dichloromethane (DCM). This catalyst permitted the acylation of **3a**–**e** at 0 °C in 30 min and delivered the unrearranged acetates **5**–**9**, respectively, in 95–98% yields. 

The results of our study are summarized in Table 1 and Table 2. The reaction of substrates **5**–**8** (1 equiv.) with AMCs **10**–**14** (1.5 equiv.) yielded 1,3,6-trisubstituted naphthalenes **15**–**18**, while precursor **9** afforded 6,8-disubstituted quinolines **19** with the same panel of nucleophiles. Naphthalene formation was promoted by K_2_CO_3_ (1.5 equiv.) in *N*,*N*-dimethylformamide (DMF) at 23 °C in 1 h, while quinolines were similarly prepared but required heating at 90 °C for 6 h. Yields were uniformly good to excellent, with the 2-fluoro-5-nitrophenyl S_N_Ar acceptor giving the most efficient ring closure, followed by the 2-fluoro-5-cyano compound and finally 2-fluoropyridine. The overall process appears to involve diastereoselective addition of the nucleophile to the double bond methylene of the MBH substrate with loss of the acetoxy group. Following this addition, deprotonation of the methine hydrogen from the added nucleophile, S_N_Ar cyclization, and elimination fuses a disubstituted benzene ring to the original aromatic nucleus. The exact sequence of events for this process is unclear and is discussed in more detail below. In the current reactions, the final aromatization occurred by the elimination of benzenesulfinic acid (PhSO_2_H) [6,16], nitrous acid (HNO_2_) [9,17], or ethoxycarbonyl (CO_2_Et, presumably by hydrolysis and decarboxylation) [9,18], all of which have precedent in the literature, although the loss of CO_2_Et in preference to the CN group was not expected. Aromatization by the elimination of CN is known [19,20], but it appears to be quite rare.

The syntheses are quite efficient, requiring only three steps and one chromatographic purification for the final products **15**–**19**. The products were isolated in good to excellent yields (75–96%) and were fully characterized by spectroscopic methods. The IR spectra indicated the presence of the expected functional groups (conjugated CO_2_Et or CN, polarized conjugated double bonds, and NO_2_). The naphthalenes showed three one-proton doublets (*J_1,3_* < 3 Hz or apparent singlets) for the isolated protons at C2, C4, and C5, a doublet of doublets (*J_1,2 and 1,3_* = large and small) for the proton at C7, and a doublet (*J_1,2_* > 8 Hz) for the proton at C8. The chemical shifts were in accordance with those expected for an electron-deficient aromatic system. The quinolines exhibited two doublets (*J_1,3_* < 3 Hz) for the protons at C2 and C4 as well as characteristic chemical shifts and couplings for the protons on C5–C7 of the system. The ^13^C-NMR were appropriate with respect to the number of carbonyl, aromatic, and aliphatic carbons. The mass spectra showed an ion corresponding to the molecular weight of the compound, and elemental analyses confirmed the formulas and purity.

**Scheme 2:**
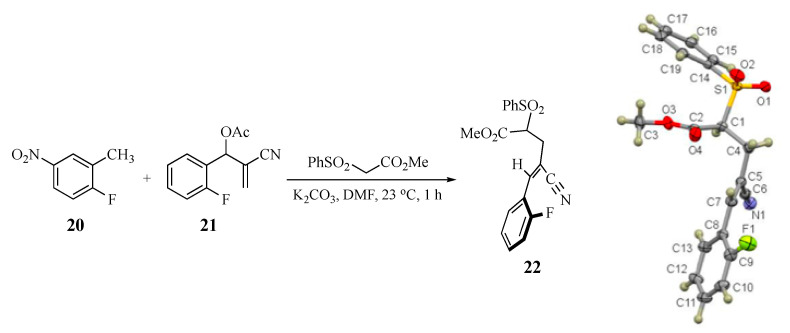
Control experiment: Selective reaction of the MBH acetate and formation of the *Z* alkene **22** (CCDC 2035022).

Two basic mechanisms are possible for this domino transformation: (1) initial S_N_Ar attack by the AMC anion on the aromatic ring, followed by conjugate addition to the side chain double bond with an elimination of acetoxy or (2) initial S_N_2′-type displacement of acetoxy from the side chain, followed by a S_N_Ar cyclization. Option 1 would unquestionably predict the final product of the reaction and would not depend on the selective formation of a single alkene from the S_N_2′ process. Option 2 would require a diastereoselective addition to give the double bond geometry that positions the active methylene fragment cis to the S_N_Ar acceptor ring. To probe this aspect of the transformation, a control experiment was performed wherein the anion of methyl phenylsulfonylacetate was generated in the presence of a mixture of 2-fluoro-5-nitrotoluene (**20**) and 2-cyano-1-(2-fluorophenyl)allyl acetate (**21**) to determine the relative reactivity of the side chain versus the aromatic ring. If the side chain reacted preferentially, we were also interested in assessing any *E*-*Z* diastereoselectivity in the allylically rearranged double bond. Under the standard conditions, a reaction occurred exclusively at the side chain of the MBH acetate and also afforded only methyl (*Z*)-4-cyano-5-(2-fluorophenyl)-2-(phenylsulfonyl)-4-pentenoate (**22**, 70%) having the added active methylene nucleophile positioned *trans* to the aromatic S_N_Ar acceptor ring. This was confirmed by an X-ray structure of adduct **22** (see Scheme 2 and the Supplementary Materials). Compound **20** (93%) was recovered unchanged from this experiment. Thus, the overall sequence must proceed via Option 2 above, followed by double bond equilibration prior to S_N_Ar ring closure.

**Figure 2:**
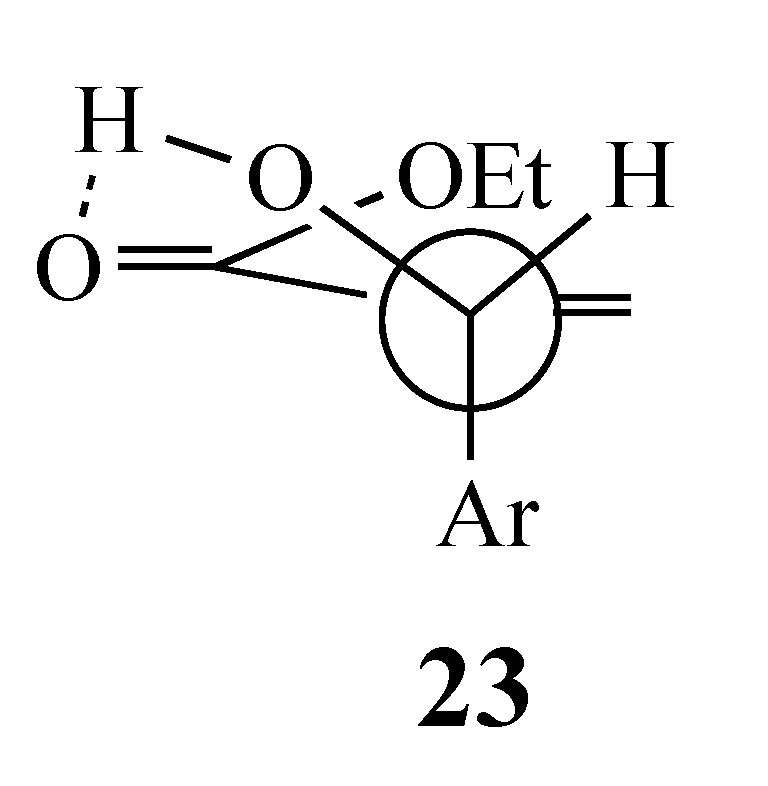
Conformation of an MBH alcohol with a stabilizing H-bond.

**Scheme 3:**
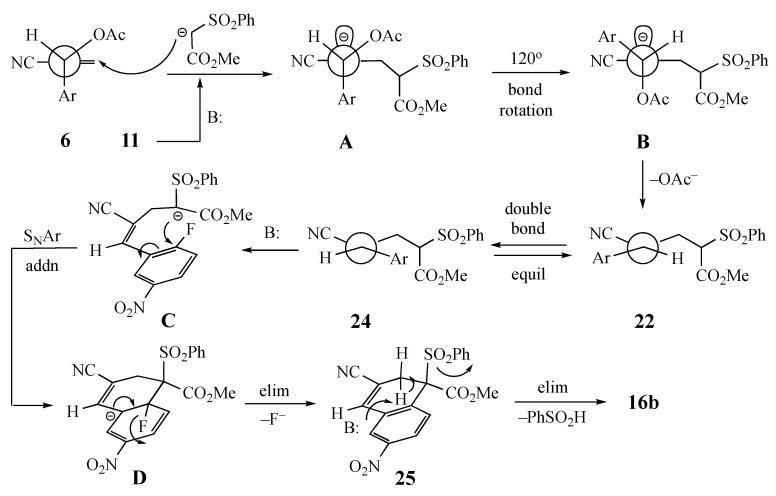
S_N_2′ mechanism leading to the formation of naphthalenes from MBH acetates.

Analysis of the reactant in the S_N_2′ reaction (Option 2 above) demands that the starting conformation minimizes the steric interaction between the aryl, the acetoxy, and the electron-withdrawing group (EWG = CO_2_Et or CN) (Scheme 2). An earlier study describing the reaction of an acrylate-derived MBH alcohol with HBr proposed conformation **23** (Figure 2), which has an additional stabilizing H-bond between the OH and the EWG, to rationalize the products [20]. This model would favor formation of the alkene that places the incoming active methylene fragment cis to the 2-fluoro-5-nitrophenyl moiety in the product. With the current substrates, H-bonding is not possible, and so the large acetoxy group was expected to orient away from the EWG (perhaps more so for CO_2_Et than CN) to minimize steric crowding (Scheme 3). Then, an initial S_N_2′ reaction involving a syn orientation of the nucleophile and leaving group invoked in some earlier work [21,22] would give an adduct with the AMC fragment trans to the S_N_Ar acceptor ring as in the aforementioned competitive reaction. Thus, in order for the cyclization to occur, the initial Michael adduct **22** must equilibrate to bring the nucleophilic center cis to the activated aromatic ring to allow cyclization by an intramolecular S_N_Ar reaction. Possible mechanisms for this isomerization are discussed below. 

In reactions at the side chain allylic moiety, there are two possible scenarios to explain the addition to the MBH acetate. The first [22] invokes an S_N_2′ process where a nucleophile attacks C1 (unsubstituted) of the allylic moiety, resulting in an accumulation of electron density on the opposite face of the molecule at C2. Then, this electron density is responsible for assisting in the departure of the acetoxy leaving group at C3. This process has been used to rationalize the reported preference for a syn orientation between the incoming nucleophile and the leaving group often seen in the reaction [23,24,25], although exceptions are known [26] with certain Nu^–^/L^–^ combinations. The second scenario [27] claims that there is no justification for a syn orientation between the nucleophile and the leaving group. In fact, in highly substituted derivatives, such as **5**–**9**, it has been suggested that the formation of a stabilized carbocation is more likely, in which case the avoidance of steric interactions guides the transformation to give the final product stereochemistry.

The S_N_2′ initiated mechanism is depicted in Scheme 3 for the reaction of **6** with **11** to give **16b**. The initial conformation of **6** would allow minimal steric interaction between the groups on the allylic side chain. In this conformation, the acetoxy substituent should hinder approach to the top face while the planar aromatic ring does not significantly block the bottom face. Introduction of the nucleophile to the bottom face would result in an accumulation of negative charge on the top face of the allylic system, as shown in **A**. Rotation of the acetoxy group 120° would position it anti to the accumulated negative charge and move the substrate 2-fluoro-5-nitrophenyl (Ar) moiety away from the newly added nucleophile to give **B**. Then, the elimination of acetoxy would deliver the *Z* alkene **22**. Subsequent equilibration of the double bond geometry to generate **24** and deprotonation of the residual active methine proton to give **C** would be followed by *ipso* attack at the fluorine-bearing aromatic carbon to afford Meisenheimer intermediate **D**. Rearomatization by loss of fluoride should then lead to **25**. After ring closure, a conformation aligned for the elimination of benzenesulfinic acid by an E2 process would afford the fused aromatic compound **16b**.

**Scheme 4:**
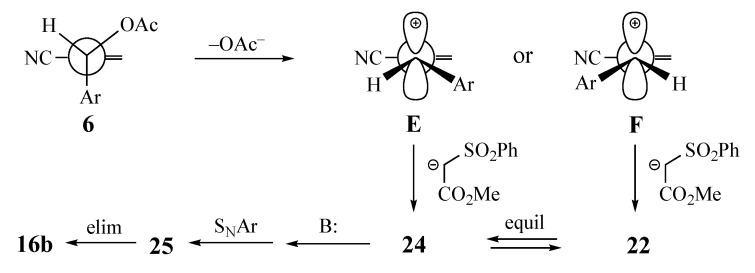
Carbocation mechanism leading to the formation of naphthalenes from MBH acetates.

The second mechanistic scenario, illustrated in Scheme 4, begins with loss of the acetoxy group to form stabilized 3-arylallylic cations **E** and **F** [27]. At this point, it may appear that addition to carbocation **F** should be easier, since the alkene terminus is less hindered. Furthermore, since the EWG should not be coplanar with the allylic cation, it should have less steric influence on the reaction, and the larger bond angles around the carbocation should permit the formation of both **E** and **F**. Addition of the active methylene nucleophile would then lead to both **22** and **24**, which could cyclize as above. In both examples cited in this paper, the alkene with the nucleophile trans to the S_N_Ar aromatic system was isolated with no contamination by the cis. This observation could disqualify this mechanism, but if **24** cyclizes very rapidly, it may not be possible to detect or isolate this short-lived intermediate.

**Scheme 5:**
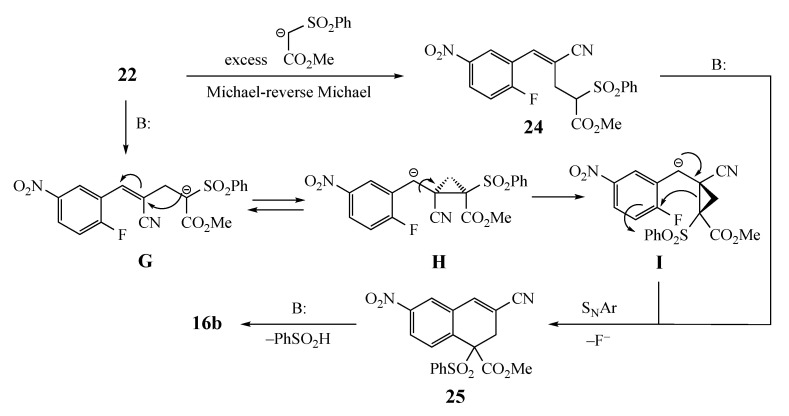
Possible mechanisms for double bond equilibration.

Two pathways can be envisioned for the equilibration of **22** to **24** that must occur before S_N_Ar cyclization: (1) a well-precedented Michael-reverse Michael process [28,29,30,31,32] involving the excess AMC or perhaps (2) an intramolecular addition–elimination reaction (Scheme 5). This alternative process would involve loss of the acidic methine proton from **22** and addition of the resulting anion **G** to the benzylic double bond to give **H**. This may be possible, since the electron-deficient aromatic ring should stabilize the benzylic anion. Then, bond rotation to give **I** and ring opening would deliver the anion needed for cyclization. Once equilibration occurs, ring closure to **25** and elimination to generate **16b** should be facile. Even if the equilibrium does not strongly favor the required alkene geometry, product formation should gradually siphon the initial adduct over to the fused aromatic target.

Addition to the substrates with formation of the *Z* alkene (EWG and added AMC trans) was confirmed by both a side reaction observed during the synthesis to form acetate **4** and a control experiment to form **15** (Scheme 2). The formation of a single product has significant precedent in the case of EWG = CO_2_Et, but it is less probable for the sterically smaller EWG = CN group [33]. Unfortunately, the ester intermediate, while a single isomer, was not a solid and could not be subjected to X-ray structure analysis. Additionally, Nuclear Overhauser enhancement (NOE) measurements were inconclusive. Nevertheless, products incorporating both EWGs formed in high yields. In cases where the initial addition yields the *Z* alkene, a reversible Michael reaction [28,29,30,31,32] or intramolecular isomerization could establish an *E*–*Z* equilibrium that would eventually cyclize and eliminate H–L to afford the aromatic products. A similar rationale can be applied to the pyridine-containing substrates that lead to quinolines. The stereoselective formation of the alkenes with the added nucleophile cis to the aromatic ring has been observed in the past with small nucleophiles [21,34,35,36,37,38,39,40]. This has been confirmed by spectral characterization, and in one instance, X-ray analysis [41]. However, our results, using large stabilized nucleophiles, appear to differ from these earlier findings.

## 3. Material and Methods

### 3.1. General Methods

Unless otherwise indicated, all reactions were carried out under dry N_2_ in oven-dried glassware. All reagents and solvents were used as received. Reactions were monitored by thin layer chromatography on silica gel GF plates (Analtech No 21521, Newark, DE, USA). Preparative separations were performed by flash chromatography on silica gel (Davisil^®^, grade 62, 60–200 mesh, Sorbent Technologies, Norcross, GA, USA) containing UV-active phosphor (Sorbent Technologies No. UV-05) slurry packed into quartz columns. Band elution for all chromatographic separations was monitored using a hand-held UV lamp (Fisher Scientific, Pittsburgh, PA, USA). Wash solutions used in work-up procedures were all aqueous. Melting points were obtained using a MEL-TEMP apparatus (Cambridge, MA, USA) and are uncorrected. FT-IR spectra were run using a Varian Scimitar FTS 800 spectrophotometer (Randolph, MA, USA) as thin films on NaCl disks. ^1^H- and ^13^C-NMR spectra were measured using a Bruker Avance 400 system (Billerica, MA, USA) in the indicated solvents at 400 MHz and 101 MHz, respectively, with (CH_3_)_4_Si as the internal standard; coupling constants (*J*) are given in Hz. Low-resolution mass spectra were obtained using a Hewlett-Packard Model 1800A GCD GC-MS system (Palo Alto, CA, USA). Details of the X-ray structure determinations are given in the Supplementary Materials. Elemental analyses (± 0.4%) were determined by Atlantic Microlabs (Norcross, GA, USA). 

### 3.2. Representative Procedure for the Synthesis of MBH Alcohols (**3a**–**e**)

To a stirred solution of the aldehyde **1a**–**c** (1 equiv.) and DABCO (1.2 equiv.) in ACN (8 mL) under N_2_ was added ethyl acrylate (**2a**) or acrylonitrile (**2b**) (2 equiv.) at 23 °C. After 24–48 h, TLC analysis (10% EtOAc/hexane) indicated the reaction was complete. The solution was added to water, and the mixture was extracted with EtOAc (3 × 25 mL). The combined organic layers were washed with 0.5 M HCl (2 × 10 mL), saturated NaHCO_3_, and saturated NaCl and then dried (Na_2_SO_4_). Removal of the solvent under vacuum resulted in pure MBH alcohols **3a**–**e**, which were used without further purification.

### 3.3. Synthesis of (E)-2-cyano-3-(2-fluoro-5-nitrophenyl)allyl acetate (**4**)

A solution of the MBH alcohol **3b** (1.11 g, 5 mmol) was dissolved in acetic anhydride (5.0 mL) and boiled for 6 h. The mixture was cooled, concentrated under vacuum, and the residue was dissolved in DCM (25 mL). The solution was washed with saturated NaHCO_3_ (3 × 50 mL) and water, dried (Na_2_SO_4_), and concentrated under vacuum to afford **4** (1.13 g, 4.3 mmol, 86%) as an off-white solid, m.p. 78–79 °C. IR: 2222, 1749, 1621, 1530, 1352 cm^−1^; ^1^H-NMR (400 MHz, CDCl_3_): δ 8.99 (dd, *J* = 6.3, 2.8 Hz, 1H), 8.35 (ddd, *J* = 9.0, 4.4, 2.8 Hz, 1H), 7.43 (s, 1H), 7.33 (t, *J* = 9.0 Hz, 1H), 4.22 (d, *J* = 1.3 Hz, 2H), 2.19 (s, 3H); ^13^C-NMR (101 MHz, CDCl_3_): δ 170.1, 163.5 (d, *J* = 263.3 Hz), 144.4, 135.9 (d, *J* = 5.4 Hz), 127.8 (d, *J* = 10.7 Hz), 125.4 (d, *J* = 3.7 Hz), 122.1 (d, *J* = 14.3 Hz), 116.1 (d, *J* = 24.4 Hz), 115.5, 112.4, (d, *J* = 2.1 Hz), 64.2, 20.7; MS (EI): *m/z* 264 [M]^+·^; Anal. Calcd for C_12_H_9_FN_2_O_4_: C 54.55; H, 3.43; N, 10.60. Found: C, 54.58; H, 3.45; N, 10.53. The X-ray structure of **4** (CCDC 2035023) was obtained and the thermal elipsoid plot is shown in Scheme 1 and the Supplementary Materials.

### 3.4. Representative procedure for the Synthesis of MBH Acetates (**5**–**9**)

A solution of the MBH alcohol **3a**–**d** (1 equiv.) in DCM (2 mL) was treated with acetic anhydride (1.5 equiv.) at 0 °C, followed by a 1 M solution of TMSOTf in DCM (20 μL/mmol substrate) [15]. The MBH alcohol **3e** (1 equiv.) required acetic anhydride (3 equiv.) and 1 M TMSOTf in DCM (60 μL/mmol substrate). After 30 min, TLC analysis (10% ether in hexane) showed the reaction was complete. The reaction mixture was added to saturated NaHCO_3_, and the mixture was extracted with DCM (3 × 5 mL). The organic extracts were washed with water, dried (Na_2_SO_4_), and evaporated under vacuum to afford clean MBH acetates **5**–**9**, which did not require further purification. After optimization, this procedure was scaled up by a factor of five with no significant decrease in yield. 

#### 3.4.1. Ethyl 2-(acetoxy (2-fluoro-5-nitrophenyl)methyl)acrylate (**5**)

Yield: 305 mg (0.98 mmol, 98%) as a colorless oil; IR: 1751, 1723, 1639, 1532, 1351 cm^−1^; ^1^H-NMR (400 MHz, CDCl_3_): δ 8.26 (dd, *J* = 6.0, 2.8 Hz, 1H), 8.23 (ddd, *J* = 8.9, 4.3, 2.9 Hz, 1H), 7.22 (t, *J* = 8.9 Hz, 2H), 6.91 (s, 1H), 6.54 (s, 1H), 4.18 (qd, *J* = 7.1, 1.3 Hz, 2H), 2.15 (s, 3H), 1.25 (t, *J* = 7.1 Hz, 3H); ^13^C-NMR (101 MHz, CDCl_3_): δ 169.0, 164.2, 163.7 (d, *J* = 232.5 Hz ), 144.2 (d, *J* = 2.9 Hz), 137.4, 127.5, 127.4 (d, *J* = 3.1 Hz), 126.0 (d, *J* = 10.5 Hz), 125.1 (d, *J* = 5.4 Hz), 116.9 (d, *J* = 24.5 Hz), 66.6 (d, *J* = 2.8 Hz), 61.3, 20.8 (d, *J* = 1.6 Hz), 14.0; MS: *m*/*z* 311 [M]^+·^; Anal. Calcd for C_14_H_14_FNO_6_: C, 54.02; H, 4.53; N, 4.50. Found: C, 53.94; H, 4.54; N, 4.39.

#### 3.4.2. 2-Cyano-1-(2-fluoro-5-nitrophenyl) allyl acetate (**6**)

Yield: 259 mg (0.98 mmol, 98%) as off-white crystals, m.p. 66–67 °C; IR: 2232, 1759, 1632, 1532, 1535, 1352 cm^−1^; ^1^H-NMR (400 MHz, CDCl_3_): δ 8.42 (dd, *J* = 6.1, 2.8 Hz, 1H), 8.31 (ddd, *J* = 9.0, 4.4, 2.8 Hz, 1H), 7.29 (t, *J* = 9.0 Hz, 1H), 6.64 (s, 1H), 6.19 (s, 1H), 6.18 (s, 1H), 2.26 (s, 3H); ^13^C-NMR (101 MHz, CDCl_3_): δ 168.8, 163.1 (d, *J* = 260.1 Hz), 144.7, 133.7, 126.8 (d, *J* = 10.3 Hz), 125.3 (d, *J* = 15.1 Hz), 124.1 (d, *J* = 4.9 Hz), 120.9, 117.1 (d, *J* = 23.7 Hz), 115.2, 67.8 (d, *J* = 3.0 Hz), 20.8; MS: *m*/*z* 264 [M]^+·^; Anal. Calcd for C_12_H_9_FN_2_O_4_: C, 54.55; H, 3.43; N, 10.60. Found: C, 54.63; H, 3.45; N,10.52.

#### 3.4.3. Ethyl 2-(acetoxy(5-cyano-2-fluorophenyl)methyl)acrylate (**7**)

Yield: 285 mg (0.98 mmol, 98%) as a colorless oil; IR: 2234, 1752, 1729, 1637 cm^−1^; ^1^H-NMR (400 MHz, CDCl_3_): δ 7.69 (dd, *J* = 6.6, 2.1 Hz, 1H), 7.64 (ddd, *J* = 8.5, 4.7, 2.1 Hz, 1H), 7.19 (t, *J* = 9.3 Hz, 1H), 6.88 (s, 1H), 6.51 (s, 1H), 5.92 (s, 1H), 4.19 (q, *J* = 7.1 Hz, 2H), 2.15 (s, 3H), 1.25 (t, *J* = 7.1 Hz, 3H); ^13^C-NMR (101 MHz, CDCl_3_): δ 169.0, 164.2, 162.6 (d, *J* = 260.3 Hz), 137.6, 134.4 (d, *J* = 9.8 Hz), 133.6 (d, *J* = 4.8 Hz), 127.7 (d, *J* = 14.9 Hz), 127.3, 117.8, 117.2 (d, *J* = 23.4 Hz), 108.7 (d, *J* = 4.0 Hz), 66.6 (d, *J* = 2.9 Hz), 61.3, 20.8, 14.0; MS: *m*/*z* 291 [M]^+·^; Anal. Calcd for C_15_H_14_FNO_4_: C, 61.85; H, 4.84; N, 4.81. Found: C, 61.77; H, 4.86; N, 4.85.

#### 3.4.4. 2-Cyano-1-(5-cyano-2-fluorophenyl)allyl acetate (**8**)

Yield: 259 mg (0.98 mmol, 98%) as off-white crystals, m.p. 74–75 °C; IR: 2255, 2235, 1757, 1613 cm^−1^; ^1^H-NMR (400 MHz, CDCl_3_): δ 7.86 (dd, *J* = 6.6, 2.1 Hz, 1H), 7.72 (ddd, *J* = 8.4, 4.8, 2.1 Hz, 1H), 7.24 (t, *J* = 9.3 Hz, 1H), 6.60 (s, 1H), 6.16 (s, 1H), 6.15 (s, 1H), 2.24 (s, 3H); ^13^C-NMR (101 MHz, CDCl_3_): δ 168.8, 161.9 (d, *J* = 259.0 Hz), 135.2 (d, *J* = 9.8 Hz), 133.7, 132.3 (d, *J* = 4.3 Hz), 125.6 (d, *J* = 14.2 Hz), 120.9, 117.44 (d, *J* = 22.8 Hz), 117.41, 115.3, 109.6 (d, *J* = 4.0 Hz), 67.8 (d, *J* = 3.2 Hz), 20.8; MS: *m*/*z* 244 [M]^+·^; Anal. Calcd for C_13_H_9_FN_2_O_2_: C, 63.93; H, 3.71; N, 11.47. Found: C, 63.81; H, 3.77; N, 11.35.

#### 3.4.5. Ethyl 2-(acetoxy(2-fluoropyridin-3-yl)methyl)acrylate (**9**)

Yield: 254 mg (0.95 mmol, 95%) as a colorless oil; IR: 1758, 1727, 1637 cm^−1^; ^1^H-NMR (400 MHz, CDCl_3_): δ 8.19 (m, 1H), 7.81 (ddd, J = 9.4, 7.4, 2.0 Hz, 1H), 7.20 (ddd, J = 6.5, 4.8, 1.6 Hz, 1H), 6.81 (s, 1H), 6.50 (s, 1H), 5.92 (s, 1H), 4.18 (q, *J* = 7.1 Hz, 2H), 2.14 (s, 3H), 1.24 (t, *J* = 7.1 Hz, 3H); ^13^C-NMR (101 MHz, CDCl_3_): δ 169.1, 164.4, 161.0 (d, *J* = 241.9 Hz), 147.6 (d, *J* = 14.9 Hz), 140.1 (d, *J* = 4.3 Hz), 137.5, 127.2 (d, *J* = 1.2 Hz), 121.4 (d, *J* = 4.4 Hz), 120.5 (d, *J* = 27.9 Hz), 67.8, 61.2, 20.8, 14.0; MS: *m*/*z* 267 [M]^+·^; Anal. Calcd for C_13_H_14_FNO_4_: C, 58.42; H, 5.28; N, 5.24. Found: C, 58.33; H, 5.25; N, 5.16. 

### 3.5. Representative Procedure for the Synthesis of Naphthalene and Quinoline Analogs Using MBH Acetates and Active Methylene Compounds

A 50 mL, round-bottomed flask equipped with a condenser, stir bar, and N_2_ inlet, was charged with an MBH acetate (1 mmol) in DMF (2 mL). The corresponding active methylene compound (1.5 mmol) in DMF (1 mL) and K_2_CO_3_ (207 mg, 1.5 mmol) were added at room temperature with continued stirring. For naphthalenes **15**–**18**, the reaction was complete in 1 h. For quinolines **19**, the reaction was stirred at room temperature for 1 h and gradually heated to 90 °C with stirring for 6 h. In each case, TLC analysis (30% EtOAc in hexane) indicated the reaction was complete. The solution was poured into de-ionized water (15 mL), and the mixture was extracted with EtOAc (3 × 25 mL). The combined organic layers were washed with saturated NaCl and dried (Na_2_SO_4_). Removal of the solvent under vacuum gave the crude product, which was purified by silica gel column chromatography to afford the pure naphthalene/quinoline derivatives.

#### 3.5.1. Ethyl 4-cyano-7-nitro-2-naphthoate (**15a**) from **5** and ethyl cyanoacetate (**10**)

Yield: 243 mg (0.90 mmol, 90%) as an off-white solid, m.p. 77–79 °C; IR: 1716, 1536, 1349 cm^−1^; ^1^H-NMR (400 MHz, CDCl_3_): δ 8.91 (d, J = 2.3 Hz, 2H), 8.60 (d, *J* = 1.6 Hz, 1H), 8.48 (dd, *J* = 9.2, 2.3 H_Z,_ 1H), 8.36 (d, *J* = 9.2 Hz, 1H), 4.42 (q, *J* = 7.1 Hz, 2H), 1.39 (t, *J* = 7.1 Hz, 3H); ^13^C-NMR: δ 163.9, 147.1, 136.8, 136.2, 135.2, 131.5, 129.7, 127.4, 126.2, 124.0, 116.1, 111.6, 62.4, 14.3; MS (EI): *m*/*z* 270; Anal. Calcd for C_14_H_10_N_2_O_4_: C, 62.22; H, 3.73; N, 10.37. Found: C, 62.26; H, 3.76; N, 10.28.

#### 3.5.2. 3-Ethyl 1-methyl 6-nitronaphthalene-1,3-dicarboxylate (**15b**) from **5** and methyl phenylsulfonylacetate (**11**)

Yield: 276 mg (0.91 mmol, 91%) as an off-white solid, m.p. 203–204 °C; IR: 1707, 1527, 1350 cm^−1^; ^1^H-NMR (400 MHz, CDCl_3_): δ 9.20 (d, *J* = 9.5 Hz, 1H), 8.95–8.90 (complex, 3H), 8.45 (dd, *J* = 9.5, 2.5 Hz_,_ 1H), 4.51 (q, *J* = 7.1 Hz, 2H), 4.07 (s, 3H), 1.49 (t, *J* = 7.1 Hz, 3H); ^13^C-NMR (101 MHz, CDCl_3_): δ 166.5, 164.9, 146.0, 137.0, 135.7, 133.0, 132.3, 129.0, 128.2, 127.9, 125.9, 122.9, 62.0, 52.8, 14.4; MS (EI): *m*/*z* 303 [M]^+·^; Anal. Calcd for C_15_H_13_NO_6_: C, 59.41; H, 4.32; N, 4.62. Found: C, 59.48; H, 4.30; N, 4.51.

#### 3.5.3. Diethyl 6-nitronaphthalene-1,3-dicarboxylate (**15c**) from **5** and ethyl nitroacetate (**12**)

Yield: 298 mg (0.94 mmol, 94%) as an off-white solid, m.p. 181–182 °C; IR: 1708, 1526, 1350 cm^−1^; ^1^H-NMR (400 MHz, CDCl_3_): δ 9.19 (d, *J* = 9.5 Hz, 1H), 8.95-8.90 (complex, 3H), 8.44 (dd, *J* = 9.5, 2.4 H_Z,_ 1H), 4.53 (q, *J* = 7.1 Hz, 2H), 4.51 (q, *J* = 7.1 Hz, 2H), 1.50 (t, *J* = 7.1 Hz, 3H), 1.49 (t, *J* = 7.1 Hz, 3H); ^13^C-NMR (101 MHz, CDCl_3_): δ 166.1, 165.0, 145.9, 136.8, 135.7, 132.8, 132.3, 129.0, 128.4, 128.2, 125.9, 122.8, 62.0, 61.9, 14.4, 14.35; MS (EI): *m*/*z* 317 [M]^+·^; Anal. Calcd for C_16_H_15_NO_6_: C, 60.57; H, 4.77; N, 4.41. Found: C, 60.63; H, 4.73; N, 4.31.

#### 3.5.4. Ethyl 4-benzoyl-7-nitro-2-naphthoate (**15d**) from **5** and 1-phenyl-2-(phenylsulfonyl)ethan-1-one (**13**)

Yield: 335 mg (0.96 mmol, 96%) as a white solid, m.p. 160–161 °C; IR: 1724, 1657, 1530, 1350 cm^−1^; ^1^H-NMR (400 MHz, CDCl_3_): δ 8.99 (d, *J* = 2.4 Hz, 1H), 8.93 (s, 1H), 8.36 (m_,_ 2H), 8.27 (d, *J* = 9.3 Hz, 1H), 7.86 (d, *J* = 7.4 Hz, 2H), 7.67 (t, *J* = 7.5 Hz, 1H), 7.51 (t, *J* = 7.7 Hz, 2H), 4.48 (q, *J* = 7.1 Hz, 2H), 1.45 (t, *J* = 7.1 Hz, 3H); ^13^C-NMR (101 MHz, CDCl_3_): δ 196.1, 165.0, 146.2, 137.2, 137.18, 135.3, 135.0, 134.1, 132.1, 130.5, 130.0, 128.9, 128.8, 127.8, 126.0, 122.5, 62.0, 14.3; MS (EI): *m*/*z* 349 [M]^+·^; Anal. Calcd for C_20_H_15_NO_5_: C, 68.76; H, 4.33; N, 4.01. Found: C, 68.67; H, 4.35; N, 3.95.

#### 3.5.5. Ethyl 4-acetyl-7-nitro-2-naphthoate (**15e**) from **5** and 1-(phenylsulfonyl)propan-2-one (**14**)

Yield: 262 mg (0.92 mmol, 92%) as a white flaky solid, m.p. 152–153 °C; IR: 1714, 1690, 1526, 1351 cm^−1^; ^1^H-NMR (400 MHz, CDCl_3_): δ 9.01 (br t, *J* = 8.2 Hz, 1H), 8.90 (m, 2H), 8.73 (br s_,_ 1H), 8.42 (br t, J = 8.4 Hz, 1H), 4.52 (q, J = 7.1 Hz, 2H), 2.84 (s, 3H), 1.49 (t, J = 7.1 Hz, 3H); ^13^C-NMR (101 MHz, CDCl_3_): δ 200.3, 165.0, 146.1, 136.6, 135.6, 134.6, 132.5, 131.3, 128.7, 128.5, 125.7, 123.2, 62.1, 29.8, 14.4; MS (EI): m/z 287 [M]^+·^; Anal. Calcd for C_15_H_13_NO_5_: C, 62.72; H, 4.56; N, 4.88. Found: C, 62.68; H, 4.55; N, 4.79.

#### 3.5.6. 6-Nitronaphthalene-1,3-dicarbonitrile (**16a**) from **6** and **10**

Yield: 178 mg (0.80 mmol, 80%) as a white solid, m.p. 224 °C (dec); IR: 2250, 1541, 1354 cm^−1^; ^1^H-NMR (400 MHz, CDCl_3_): δ 8.97 (d, J = 2.2 Hz, 1H), 8.67 (s, 1H), 8.65 (dd, J = 9.1, 2.2 Hz, 1H), 8.51 (d, J = 9.2 Hz, 1H), 8.26 (d, J = 1.6 Hz, 1H); ^13^C-NMR (101 MHz, CDCl_3_): δ 147.6, 139.7, 135.7, 135.5, 131.4, 127.9, 125.3, 125.0, 116.0, 114.9, 113.2, 112.0; MS (EI): m/z 223 [M]^+·^; Anal. Calcd for C_12_H_5_N_3_O_2_: C, 64.58; H, 2.26; N, 18.83. Found: C, 64.52; H, 2.29; N, 18.74.

#### 3.5.7. Methyl 3-cyano-6-nitro-1-naphthoate (**16b**) from **6** and **11**

Yield: 220 mg (0.86 mmol, 86%) as a white solid, m.p. 219-220 °C; IR: 2254, 1708, 1534, 1350 cm^−1^; ^1^H-NMR (400 MHz, CDCl_3_): δ 9.26 (d, *J* = 9.5 Hz, 1H), 8.89 (d, *J* = 2.4 Hz, 1H), 8.58 (s, 1H), 8.54 (d, *J* = 1.7 Hz, 1H), 8.51 (dd, *J* = 9.5, 2.4 Hz, 1H), 4.08 (s, 3H); ^13^C-NMR (101 MHz, CDCl_3_): δ 165.3, 146.5, 139.8, 135.0, 133.8, 132.2, 129.1, 128.6, 125.0, 123.8, 117.1, 111.3, 53.1; MS (EI): *m*/*z* 256 [M]^+·^; Anal. Calcd for C_13_H_8_N_2_O_4_: C, 60.94; H, 3.15; N, 10.93. Found: C, 60.87; H, 3.13; N, 10.87.

#### 3.5.8. Ethyl 3-cyano-6-nitro-1-naphthoate (**16c**) from **6** and **12**

Yield: 238 mg (0.88 mmol, 88%) as a white solid, m.p. 156–157 °C; IR: 2254, 1722, 1629, 1535, 1350 cm^−1^; ^1^H-NMR (400 MHz, CDCl_3_): δ 9.26 (d, *J* = 9.5 Hz, 1H), 8.89 (d, *J* = 2.4 Hz, 1H), 8.58 (s, 1H), 8.53 (s, 1H), 8.51 (dd, *J* = 9.5, 2.4 Hz, 1H), 4.54 (q, *J* = 7.1 Hz, 2H), 1.50 (t, *J* = 7.1 Hz, 3H); ^13^C-NMR (101 MHz, CDCl_3_): δ 164.9, 146.5, 139.6, 135.0, 133.7, 132.1, 129.5, 128.7, 125.0, 123.7, 117.2, 111.3, 62.4, 14.3; MS (EI): *m*/*z* 270 [M]^+·^; Anal. Calcd for C_14_H_10_N_2_O_4_: C, 62.22; H, 3.73; N, 10.37. Found: C, 62.18; H, 3.72; N, 10.31.

#### 3.5.9. 4-Benzoyl-7-nitro-2-naphthonitrile (**16d**) from **6** and **13**

Yield: 272 mg (0.90 mmol, 90%) as a white solid, m.p. 152–154 °C; IR: 2234, 1663, 1530, 1348 cm^−1^; ^1^H-NMR (400 MHz, CDCl_3_): δ 8.94 (d, *J* = 2.3 Hz, 1H), 8.58 (s, 1H), 8.42 (dd, *J* = 9.3, 2.3 Hz, 1H), 8.29 (d, *J* = 9.3 Hz, 1H), 7.90 (d, *J* = 1.6 Hz, 1H), 7.84 (dd, *J* = 8.4, 1.7 Hz, 2H), 7.71 (tt, *J* = 7.5, 1.4 Hz, 1H), 7.54 (t, *J* = 7.5 Hz, 2H); ^13^C-NMR (101 MHz, CDCl_3_): δ 194.7, 146.8, 138.5, 137.8, 136.6, 134.61, 134.55, 132.0, 130.5, 130.4, 129.1, 128.2, 125.0, 123.4, 117.3, 111.1; MS (EI): *m*/*z* 302 [M]^+·^; Anal. Calcd for C_18_H_10_N_2_O_3_: C, 71.52; H, 3.33; N, 9.27. Found: C, 71.47; H, 3.31; N, 9.37.

#### 3.5.10. 4-Acetyl-7-nitro-2-naphthonitrile (**16e**) from **6** and **14**

Yield: 211 mg (0.88 mmol, 88%) as a white solid, m.p. 202–204 °C; IR: 2228, 1670, 1518, 1344 cm^−1^; ^1^H-NMR (400 MHz, CDCl_3_): δ 8.99 (d, *J* = 9.5 Hz, 1H), 8.88 (d, *J* = 2.4 Hz, 1H), 8.56 (s, 1H), 8.50 (dd, *J* = 9.5, 2.4 Hz, 1H), 8.25 (d, *J* = 1.7 Hz, 1H), 2.82 (s, 3H); ^13^C-NMR (101 MHz, CDCl_3_): δ 198.9, 146.6, 139.4, 136.8, 133.8, 132.3, 131.7, 128.8, 124.8, 124.0, 117.2, 111.1, 29.8; MS (EI): *m*/*z* 240 [M]^+·^; Anal. Calcd for C_13_H_8_N_2_O_3_: C, 65.00; H, 3.36; N, 11.66. Found: C, 64.93; H, 3.33; N, 11.59.

#### 3.5.11. Ethyl 4,7-dicyano-2-naphthoate (**17a**) from **7** and **10**

Yield: 200 mg (0.80 mmol, 80%) as a white solid, m.p. 209–210 °C; IR: 2229, 1720 cm^−1^; ^1^H-NMR (400 MHz, CDCl_3_): δ 8.87 (s, 1H), 8.66 (d, *J* = 1.5 Hz, 1H), 8.45 (s, 1H), 8.41 (d, *J* = 8.7 Hz, 1H), 7.96 (dd, *J* = 8.7, 1.5 Hz, 1H), 4.51 (q, *J* = 7.1 Hz, 2H), 1.48 (t, *J* = 7.1 Hz, 3H); ^13^C-NMR (101 MHz, CDCl_3_): δ 164.0, 135.7, 135.6, 135.2, 134.8, 131.5, 131.2, 129.4, 126.8, 117.7, 116.1, 112.5, 111.5, 62.4, 14.3; MS (EI): *m*/*z* 250 [M]^+·^; Anal. Calcd for C_15_H_10_N_2_O_2_: C, 71.99; H, 4.03; N, 11.19. Found: C, 72.04; H, 4.06; N, 11.12.

#### 3.5.12. 3-Ethyl 1-methyl 6-cyanonaphthalene-1,3-dicarboxylate (**17b**) from **7** and **11**

Yield: 232 mg (0.82 mmol, 82%) as a white solid, m.p. 181–182 °C; IR: 2224, 1707 cm^−1^; ^1^H-NMR (400 MHz, CDCl_3_): δ 9.13 (d, *J* = 9.0 Hz, 1H), 8.89 (d, *J* = 1.8 Hz, 1H), 8.77 (s, 1H), 8.38 (d, *J* = 0.9 Hz, 1H), 7.83 (dd, *J* = 9.0, 1.6 Hz, 1H), 4.50 (q, *J* = 7.1 Hz, 2H), 4.05 (s, 3H), 1.48 (t, *J* = 7.1 Hz, 3H); ^13^C-NMR (101 MHz, CDCl_3_): δ 166.5, 165.0, 135.62, 135.55, 134.5, 132.4, 132.2, 130.1, 128.6, 127.8, 127.6, 118.3, 111.0, 61.9, 52.7, 14.4; MS (EI): *m*/*z* 283 [M]^+·^; Anal. Calcd for C_16_H_13_NO_4_: C, 67.84; H, 4.63; N, 4.94. Found: C, 67.76; H, 4.62; N, 4.97.

#### 3.5.13. Diethyl 6-cyanonaphthalene-1,3-dicarboxylate (**17c**) from **7** and **12**

Yield: 247 mg (0.82 mmol, 83%) as a white solid, m.p. 159–160 °C; IR: 2223, 1714 cm^−1^; ^1^H-NMR (400 MHz, CDCl_3_): δ 9.12 (d, J = 9.1 Hz, 1H), 8.88 (d, *J* = 1.9 Hz, 1H), 8.77 (s, 1H), 8.37 (s, 1H), 7.83 (dd, J = 9.0, 1.5 Hz, 1H), 4.52 (q, *J* = 7.1 Hz, 2H), 4.50 (q, *J* = 7.1 Hz, 2H), 1.49 (t, *J* = 7.1 Hz, 3H), 1.48 (t, *J* = 7.1 Hz, 3H); ^13^C-NMR (101 MHz, CDCl_3_): δ 166.1, 165.1, 135.6, 135.4, 134.5, 132.21, 132.18, 130.1, 128.6, 128.3, 127.6, 118.3, 110.9, 61.9, 61.8, 14.36, 14.35; MS (EI): *m*/*z* 297 [M]^+·^; Anal. Calcd for C_17_H_15_NO_4_: C, 68.68; H, 5.09; N, 4.71. Found: C, 68.62; H, 5.09; N, 4.63.

#### 3.5.14. Ethyl 4-benzoyl-7-cyano-2-naphthoate (**17d**) from **7** and **13**

Yield: 280 mg (0.85 mmol, 85%) as a white solid, m.p. 144–145 °C; IR: 2227, 1722, 1656 cm^−1^; ^1^H-NMR (400 MHz, CDCl_3_): δ 8.79 (s, 1H), 8.43 (s, 1H), 8.31 (s, 1H), 8.21 (d, *J* = 8.8 Hz, 1H), 7.85 (d, *J* = 7.7 Hz, 2H), 7.74 (d, *J* = 8.8 Hz, 1H), 7.66 (t, *J* = 7.5 Hz, 1H), 7.51 (t, *J* = 7.6 Hz, 2H), 4.47 (q, *J* = 7.1 Hz, 2H), 1.44 (t, *J* = 7.1 Hz, 3H); ^13^C-NMR (101 MHz, CDCl_3_): δ 196.1, 165.1, 137.24, 137.15, 135.6, 134.2, 134.1, 133.6, 132.1, 130.4, 129.7, 129.4, 128.8, 128.5, 127.3, 118.3, 111.2, 61.9, 14.3; MS (EI): *m*/*z* 329 [M]^+·^; Anal. Calcd for C_21_H_15_NO_3_: C, 76.58; H, 4.59; N, 4.25. Found: C, 76.60; H, 4.63; N, 4.17.

#### 3.5.15. Ethyl 4-acetyl-7-cyano-2-naphthoate (**17e**) from **7** and **14**

Yield: 224 mg (0.84 mmol, 84%) as a white solid, m.p. 134–135 °C; IR: 2229, 1717, 1678 cm^−1^; ^1^H-NMR (400 MHz, CDCl_3_): δ 8.95 (d, *J* = 9.0 Hz, 1H), 8.76 (s, 1H), 8.68 (t, *J* = 1.6 Hz, 1H), 8.37 (s, 1H), 7.83 (dt, *J* = 8.9, 1.8 Hz, 1H), 4.51 (q, *J* = 7.1 Hz, 2H), 2.82 (s, 3H), 1.49 (t, *J* = 7.1 Hz, 3H); ^13^C-NMR (101 MHz, CDCl_3_): δ 200.4, 165.1, 135.5, 135.4, 135.3, 133.5, 132.4, 130.7, 130.5, 128.4, 127.9, 118.2, 111.2, 62.0, 29.8, 14.4; MS (EI): *m*/*z* 267 [M]^+·^; Anal. Calcd for C_16_H_13_NO_3_: C, 71.90; H, 4.90; N, 5.24. Found: C, 71.84; H, 4.93; N, 5.28.

#### 3.5.16. Naphthalene-1,3,6-tricarbonitrile (**18a**) from **8** and **10**

Yield: 152 mg (0.75 mmol, 75%) as a white solid, m.p. 206–207 °C; IR: 2232 cm^−1^; ^1^H-NMR: δ 8.53 (br s, 1H), 8.45 (d, *J* = 8.7 Hz, 1H), 8.42 (br s, 1H), 8.22 (d, *J* = 1.6 Hz, 1H), 8.04 (dd, *J* = 8.7, 1.6 Hz, 1H); ^13^C-NMR (101 MHz, CDCl_3_): δ 138.5, 135.2, 134.8, 134.4, 132.2, 131.3, 127.2, 117.1, 116.2, 114.8, 113.8, 113.1, 111.7; MS (EI): *m*/*z* 203 [M]^+·^; Anal. Calcd for C_13_H_5_N_3_: C, 76.84; H, 2.48; N, 20.68. Found: C, 76.89; H, 2.51; N, 20.57.

#### 3.5.17. Methyl 3,6-dicyano-1-naphthoate (**18b**) from **8** and **11**

Yield: 184 mg (0.78 mmol, 78%) as an off-white solid, m.p. 235–236 °C; IR: 2228, 1719 cm^−1^; ^1^H-NMR (400 MHz, CDCl_3_): δ 9.18 (d, *J* = 9.0 Hz, 1H), 8.49 (s, 1H), 8.45 (s, 1H), 8.34 (s, 1H), 7.91 (d, *J* = 8.9 Hz, 1H), 4.10 (s, 3H); ^13^C-NMR (101 MHz, CDCl_3_): δ 165.3, 138.5, 134.6, 133.8, 133.3, 132.0, 131.1, 129.0, 127.9, 117.7, 117.2, 112.2, 110.9, 53.1; MS (EI): *m*/*z* 236 [M]^+·^; Anal. Calcd for C_14_H_8_N_2_O_2_: C, 71.18; H, 3.41; N, 11.86. Found: C, 71.09; H, 3.43; N, 11.79.

#### 3.5.18. Ethyl 3,6-dicyano-1-naphthoate (**18c**) from **8** and **12**

Yield: 200 mg (0.80 mmol, 80%) as a white solid, m.p. 185–186 °C; IR: 2228, 1704 cm^−1^; ^1^H-NMR (400 MHz, CDCl_3_): δ 9.18 (d, *J* = 9.0 Hz, 1H), 8.48 (s, 1H), 8.44 (s, 1H), 8.34 (s, 1H), 7.90 (d, *J* = 9.0 Hz, 1H), 4.53 (q, *J* = 7.1 Hz, 2H), 1.49 (t, *J* = 7.1 Hz, 3H); ^13^C-NMR: δ 164.9, 138.3, 134.6, 133.9, 133.2, 132.0, 131.0, 129.3, 127.9, 117.8, 117.3, 112.1, 110.9, 62.3, 14.3; MS (EI): *m*/*z* 250 [M]^+·^; Anal. Calcd for C_15_H_10_N_2_O_2_: C, 71.99; H, 4.03; N, 11.19. Found: C, 72.05; H, 4.04; N, 11.07.

#### 3.5.19. 4-Benzoylnaphthalene-2,7-dicarbonitrile (**18d**) from **8** and **13**

Yield: 280 mg (0.80 mmol, 80%) as a white solid, m.p. 170–171 °C; IR: 2232, 1664 cm^−1^; ^1^H-NMR (400 MHz, CDCl_3_): δ 8.44 (s, 1H), 8.39 (s, 1H), 8.23 (d, *J* = 8.8 Hz, 1H), 7.88-7.77 (complex, 4H), 7.70 (t, *J* = 7.5 Hz, 1H), 7.53 (t, *J* = 7.7 Hz, 2H); ^13^C-NMR (101 MHz, CDCl_3_): δ 194.7, 138.4, 136.6, 136.5, 134.59, 134.55, 133.4, 131.9, 130.6, 130.4, 130.0, 129.0, 127.6, 117.7, 117.4, 112.4, 110.7; MS (EI): *m*/*z* 282 [M]^+·^; Anal. Calcd for C_19_H_10_N_2_O: C, 80.84; H, 3.57; N, 9.92. Found: C, 80.83; H, 3.54; N, 9.86.

#### 3.5.20. 4-Acetylnaphthalene-2,7-dicarbonitrile (**18e**) from **8** and **14**

Yield: 224 mg (0.79 mmol, 79%) as a white solid, m.p. 215–216 °C; IR: 2227, 1686 cm^−1^; ^1^H-NMR (400 MHz, CDCl_3_): δ 8.91 (d, *J* = 9.0 Hz, 1H), 8.43 (s, 1H), 8.34 (d, *J* = 1.7 Hz, 1H), 8.20 (d, *J* = 1.6 Hz, 1H), 7.89 (dd, *J* = 9.0, 1.7 Hz, 1H), 2.82 (s, 3H); ^13^C-NMR (101 MHz, CDCl_3_): δ 199.0, 138.1, 136.7, 134.4, 132.7, 132.2, 131.3, 131.2, 128.1, 117.7, 117.3, 112.4, 110.7, 29.8; MS (EI): *m*/*z* 220 [M]^+·^; Anal. Calcd for C_14_H_8_N_2_O: C, 76.35; H, 3.66; N, 12.72. Found: C, 76.30; H, 3.63; N, 12.67.

#### 3.5.21. Ethyl 8-cyanoquinoline-6-carboxylate (**19a**) from **9** and **10**

Yield: 170 mg (0.75 mmol, 75%) as a yellow solid, m.p. 142–144 °C; IR: 2233, 1722 cm^−1^; ^1^H-NMR (400 MHz, CDCl_3_): δ 9.20 (m, 1H), 8.81 (s, 1H), 8.73 (d, *J* = 1.7 Hz, 1H), 8.38 (dd, *J* = 8.3, 1.7 Hz, 1H), 7.65 (dd, *J* = 8.3, 4.3 Hz, 1H), 4.50 (q, *J* = 7.1 Hz, 2H), 1.48 (q, *J* = 7.1 Hz, 3H); ^13^C-NMR) (101 MHz, CDCl_3_): δ 164.3, 154.4, 149.0,137.8, 135.3, 135.2, 128.3, 127.5, 123.5, 116.5, 113.8, 62.2, 14.3; MS (EI): *m*/*z* 226 [M]^+·^; Anal. Calcd for C_13_H_10_N_2_O_2_: C, 69.02; H, 4.46; N, 12.38. Found: C, 69.07; H, 4.49; N, 12.27.

#### 3.5.22. 6-Ethyl 8-methyl quinoline-6,8-dicarboxylate (**19b**) from **9** and **11**

Yield: 212 mg (0.82 mmol, 82%) as an off-white solid, m.p. 75–77 °C; IR: 1722 cm^−1^; ^1^H-NMR (400 MHz, CDCl_3_): δ 9.14 (dd, *J* = 4.2, 1.8 Hz, 1H), 8.70 (d, *J* = 2.0 Hz, 1H), 8.62 (d, *J* = 2.0 Hz, 1H), 8.31 (dd, *J* = 8.4, 1.8 Hz, 1H), 7.54 (dd, *J* = 8.3, 4.2 Hz, 1H), 4.48 (q, *J* = 7.1 Hz, 2H), 4.08 (s, 3H), 1.46 (t, *J* = 7.1 Hz, 3H); ^13^C-NMR (101 MHz, CDCl_3_): δ 167.6, 165.3, 153.5, 147.2, 137.6, 133.9, 132.0, 129.9, 127.8, 127.7, 122.3, 61.8, 52.9, 14.4; MS (EI): *m*/*z* 259 [M]^+·^; Anal. Calcd for C_14_H_13_NO_4_: C, 64.86; H, 5.05; N, 5.40. Found: C, 64.80; H, 5.03; N, 5.37.

#### 3.5.23. Ethyl 8-benzoylquinoline-6-carboxylate (**19d**) from **9** and **13**

Yield: 244 mg (0.80 mmol, 80%) as a light yellow solid, m.p. 146–147 °C; IR: 1718, 1671 cm^−1^; ^1^H-NMR (400 MHz, CDCl_3_): δ 8.93 (d, J = 4.2 Hz, 1H), 8.72 (d, *J* = 1.9 Hz, 1H), 8.36-8.30 (complex, 2H), 7.84 (d, J = 7.3 Hz, 2H), 7.57 (t, *J* = 7.3 Hz, 1H), 7.50 (dd, J = 8.4, 4.2 Hz, 1H), 7.42 (t, *J* = 7.7 Hz, 2H), 4.46 (q, *J* = 7.1 Hz, 2H), 1.44 (t, *J* = 7.1 Hz, 3H); ^13^C-NMR (101 MHz, CDCl_3_): δ 197.1, 165.5, 152.8, 147.9, 139.8, 137.4, 137.3, 133.5, 132.4, 130.2, 128.5, 128.0, 127.62, 127.55, 122.4, 61.7, 14.4; MS (EI): *m*/*z* 305 [M]^+·^; Anal. Calcd for C_19_H_15_NO_3_: C, 74.74; H, 4.95; N, 4.59. Found: C, 74.71; H, 4.95; N, 4.52.

#### 3.5.24. Ethyl 8-acetylquinoline-6-carboxylate (**19e**) from **9** and **14**

Yield: 185 mg (0.76 mmol, 76%) as a white crystals, m.p. 103–104 °C; IR: 1704, 1689 cm^−1^; ^1^H-NMR (400 MHz, CDCl_3_): δ 9.06 (dd, J = 4.2, 1.8 Hz, 1H), 8.69 (d, *J* = 1.9 Hz, 1H), 8.50 (d, *J* = 1.9 Hz, 1H), 8.32 (dd, *J* = 8.4, 1.8 Hz, 1H), 7.54 (dd, *J* = 8.3, 4.2 Hz, 1H), 4.47 (q, *J* = 7.1 Hz, 2H), 2.94 (s, 3H), 1.46 (t, *J* = 7.1 Hz, 3H); ^13^C-NMR (101 MHz, CDCl_3_): δ 203.2, 165.5, 152.4, 147.2, 140.1, 137.6, 133.7, 128.6, 128.1, 127.7, 122.2, 61.7, 32.6, 14.4; MS (EI): *m*/*z* 243 [M]^+·^; Anal. Calcd for C_14_H_13_NO_3_: C, 69.12; H, 5.39; N, 5.76. Found: C, 69.06; H, 5.37; N, 5.69.

### 3.6. Competitive Reaction Control Experiment. Formation of Methyl (Z)-4-cyano-5-(2-fluorophenyl)-2-(phenylsulfonyl)-4-pentenoate (**22**)

A 50-mL, round-bottomed flask equipped with a condenser, stir bar, and N_2_ inlet was charged with a 2-fluoro-5-nitrotoluene (**20**, 155 mg, 1 mmol) and 2-cyano-1-(2-fluorophenyl)allyl acetate (**21**, 219 mg, 1 mmol) in DMF (2 mL) under N_2_. Methyl phenylsulfonylacetate (321 mg, 1.5 mmol) and K_2_CO_3_ (207 mg, 1.5 mmol) were added at room temperature with stirring. TLC analysis (20% EtOAc in hexane) indicated that the reaction was complete in 1 h. The solution was poured into de-ionized water (15 mL), and the mixture was extracted with EtOAc (3 × 25 mL). The combined organic layers were washed with saturated NaCl and dried (Na_2_SO_4_). Removal of the solvent under vacuum gave the crude product, which was purified by silica gel column chromatography to afford **20** (144 mg, 93%) and **22** (261 mg, 0.7 mmol, 70%) as a white solid, m.p. 95–97 °C; IR: 2215, 1744, 1637, 1149 cm^−1^; ^1^H-NMR (400 MHz, CDCl_3_): δ 7.98 (td, *J* = 7.8, 1.7 Hz, 1H), 7.92 (d, *J* = 7.8 Hz, 2H), 7.74 (tt, *J* = 7.5, 1.9 Hz_,_ 1H), 7.62 (t, *J* = 8.2 Hz, 2H), 7.44–7.36 (complex, 1H), 7.31 (s, 1H), 7.20 (t, *J* = 7.8 Hz, 1H), 7.10 (t, *J* = 9.5 Hz, 1H), 4.34 (dd, *J* = 10.7, 4.4 Hz, 1H), 3.67 (s, 3H), 3.20 (dd, *J* = 14.3, 4.4 Hz, 1H), 3.14 (dd, *J* = 14.3, 10.7 Hz, 1H); ^13^C-NMR (101 MHz, CDCl_3_): δ 164.9, 160.4 (d, *J* = 253.0 Hz), 139.3 (d, *J* = 6.5 Hz), 136.8, 134.8, 132.6 (d, *J* = 8.7 Hz), 129.4, 129.2, 128.3 (d, *J* = 1.6 Hz), 124.6 (d, *J* = 3.7 Hz), 121.1 (d, *J* = 11.7 Hz), 117.0, 115.8 (d, *J* = 21.7 Hz), 107.2 (d, *J* = 2.0 Hz), 68.7, 53.3, 33.0; MS (EI): *m/z* 373 [M]^+·^; Anal. Calcd for C_19_H_16_FNO_4_S: C, 61.12; H, 4.32; N, 3.75. Found: C, 61.22; H, 4.25; N, 3.68. The X-ray structure for compound **22** (CCDC 2035022) and the thermal elipsoid plot is shown in Scheme 2 and the Supplementary Materials.

## 4. Conclusions

We have investigated the synthesis of naphthalenes and quinolines from Morita–Baylis–Hillman acetates and active methylene compounds promoted by K_2_CO_3_ in DMF. The formation of naphthalenes occurs at 23 °C, while quinolines required heating to 90 °C. Substrates for the naphthalenes were MBH acetates bearing 2-fluoroaromatic rings activated toward S_N_Ar ring closure by C5 NO_2_ or CN groups. Quinoline precursors were activated only by the electron-withdrawing nitrogen in a 2-fluoropyridine ring. The transformation most likely involves a domino S_N_2′-S_N_Ar process. A control experiment indicated that the initial reaction occurs by an S_N_2′-type substitution of the side chain acetate to yield the alkene having the aromatic ring *trans* to the aromatic S_N_Ar acceptor ring. Thus, under the reaction conditions, a reversible Michael addition or an intramolecular addition–elimination must occur to equilibrate the geometry of the double bond to yield the alkene isomer needed for ring closure. The isolation of intermediates from the reaction disputes an earlier report that the reaction directly delivers the alkene needed for cyclization. Once equilibrated, subsequent deprotonation of the active methine proton, S_N_Ar ring closure, and the elimination of SO_2_Ph, NO_2_, or CO_2_Et then aromatizes the product. Much of the selectivity appears to be guided by steric considerations. The loss of SO_2_Ph or NO_2_ in the aromatization process has good precedent in the literature, but the loss of CO_2_Et in preference to CN was unexpected. Good to excellent yields were isolated for all examples.

## Figures and Tables

**Table 1:**
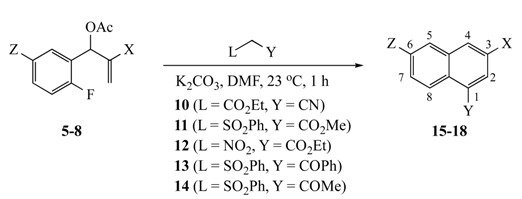
Formation of naphthalenes.

Substrate	AMC ^a^	L ^b^	X	Y	Z	Pdt (%Yield)
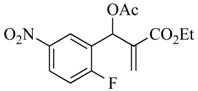 **5**	**10**	CO_2_Et	CO_2_Et	CN	NO_2_	**15a** (90)
	**11**	SO_2_Ph	CO_2_Et	CO_2_Me	NO_2_	**15b** (91)
	**12**	NO_2_	CO_2_Et	CO_2_Et	NO_2_	**15c** (94)
	**13**	SO_2_Ph	CO_2_Et	COPh	NO_2_	**15d** (96)
	**14**	SO_2_Ph	CO_2_Et	COMe	NO_2_	**15e** (92)
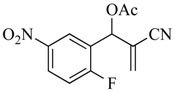 **6**	**10**	CO_2_Et	CN	CN	NO_2_	**16a** (80)
	**11**	SO_2_Ph	CN	CO_2_Me	NO_2_	**16b** (86)
	**12**	NO_2_	CN	CO_2_Et	NO_2_	**16c** (88)
	**13**	SO_2_Ph	CN	COPh	NO_2_	**16d** (90)
	**14**	SO_2_Ph	CN	COMe	NO_2_	**16e** (88)
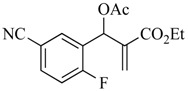 **7**	**10**	CO_2_Et	CO_2_Et	CN	CN	**17a** (80)
	**11**	SO_2_Ph	CO_2_Et	CO_2_Me	CN	**17b** (82)
	**12**	NO_2_	CO_2_Et	CO_2_Et	CN	**17c** (83)
	**13**	SO_2_Ph	CO_2_Et	COPh	CN	**17d** (85)
	**14**	SO_2_Ph	CO_2_Et	COMe	CN	**17e** (84)
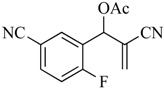 **8**	**10**	CO_2_Et	CN	CN	CN	**18a** (75)
	**11**	SO_2_Ph	CN	CO_2_Me	CN	**18b** (78)
	**12**	NO_2_	CN	CO_2_Et	CN	**18c** (80)
	**13**	SO_2_Ph	CN	COPh	CN	**18d** (80)
	**14**	SO_2_Ph	CN	COMe	CN	**18e** (79)

^a^ AMC = active methylene compound. ^b^ L = leaving group.

**Table 2:**
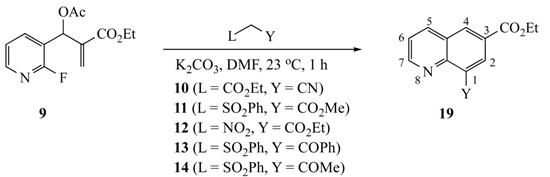
Formation of quinolines.

Substrate	AMC ^a^	L ^b^	Y	Pdt (%Yield)
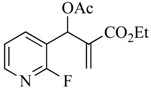 **9**	**10**	CO_2_Et	CN	**19a** (75)
	**11**	SO_2_Ph	CO_2_Me	**19b** (82)
	**12**	NO_2_	CO_2_Et	ND ^c^
	**13**	SO_2_Ph	COPh	**19d** (80)
	**14**	SO_2_Ph	COMe	**19e** (76)

^a^ AMC = active methylene compound. ^b^ L = leaving group. ^c^ ND = not done.

## Supplementary Materials

The following are available online, Copies of ^1^H-NMR and ^13^C-NMR spectra for all compounds and tables of crystal data for compounds **4** and **22**.
